# Scarlet Fever Outbreak, Hong Kong, 2011

**DOI:** 10.3201/eid1810.120062

**Published:** 2012-10

**Authors:** Eric H.Y. Lau, Hiroshi Nishiura, Benjamin J. Cowling, Dennis K.M. Ip, Joseph T. Wu

**Affiliations:** The University of Hong Kong School of Public Health, Hong Kong Special Administrative Region, People’s Republic of China (E.H.Y. Lau, H. Nishiura, B.J. Cowling, D.K.M. Ip, J.T. Wu);; and Japan Science and Technology Agency, Saitama, Japan (H. Nishiura)

**Keywords:** scarlet fever, group A streptococcus, bacteria, Hong Kong, China, outbreak

**To the Editor:** Scarlet fever is a notifiable disease in Hong Kong, Guangdong Province, and Macau in the People’s Republic of China. All 3 areas reported substantial increases in cases during 2011 (Figure, panel A). In Hong Kong, individual data, including age, geographic location, date of notification, and travel history within the incubation period, were collected from all locally notified scarlet fever case-patients. As of December 31, 2011, a total of 1,535 cases (21.7 cases/100,000 population) were reported, which was ≈10× higher than the average number of annual cases reported during the preceding 10 years (*1*). Of those, 730 cases were laboratory confirmed; 46 cases were imported; and 2 cases, 1 each in a 7-year-old girl and a 5-year-old boy co-infected with chickenpox, resulted in death (*2*).

**Figure:**
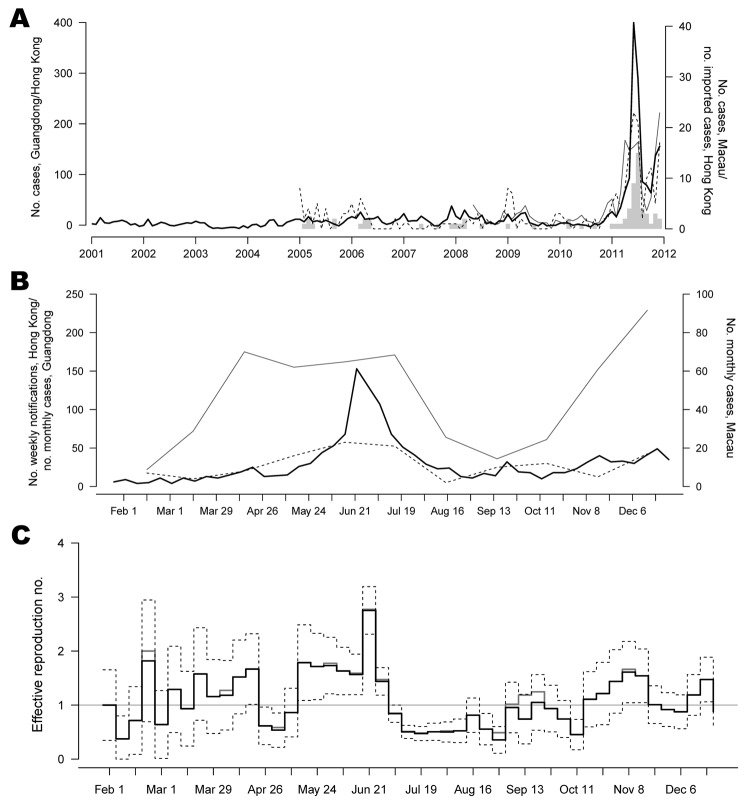
Trends in scarlet fever during outbreak in Hong Kong, Guangdong, and Macau, People’s Republic of China, 2011. A) Monthly scarlet fever notifications in Hong Kong, Guangdong (data obtained from Department of Health Guangdong Province, www.gdwst.gov.cn/a/yiqingxx), and Macau (data obtained from Health Bureau, Government of the Macau Special Administrative Region (www.ssm.gov.mo/news/content/ch/1005/statistic.aspx). Vertical tick marks indicate January of each year. Data from Guangdong and Macau were available beginning in 2005. Black line indicates data from Hong Kong; gray line, data from Guangdong; broken line, data from Macau; gray bar, number of imported cases in Hong Kong, 2005-2011. B) Weekly notifications of scarlet fever cases in Hong Kong and monthly notifications in Guangdong and Macau. Black line indicates data from Hong Kong; gray line, data from Guangdong; broken line, data from Macau. C) Estimated instantaneous reproduction number (R_t_) and 95% pointwise confidence intervals (CIs) based on scarlet fever notifications in Hong Kong, February–December, 2011. Black line indicates estimate calculated by grouping patients with unknown importation status with patients with imported cases; gray line, estimate calculated by grouping patients with unknown importation status with local case-patients; broken lines, the upper and lower limits of the 95% CIs for R_t_. For better presentation, CIs are shown only for the former estimates. Horizontal line indicates the critical value of R_t_, under which transmission of disease will not be sustainable.

Group A *Streptococcus* (GAS), the bacterium that causes scarlet fever, is mainly transmitted by direct contact with saliva and nasal fluids from infected persons (*3*). Many children can also carry GAS or be asymptomatically infected (*4*). A recent study in China showed that GAS is commonly resistant to macrolides and tetracycline but sensitive to penicillin, chloramphenicol, cefradine, and ofloxacin (*5*). In Hong Kong, GAS *emm* type 12 dominated among the isolates cultured during 2011 (*6*). Most of the cases reported were in children <10 years of age (range 1 month–51 years; median 6 years [interquartile range 4–7 years]). The age distribution is similar to that reported during previous years (data not shown).

In the United Kingdom during the mid-19th century, scarlet fever epidemics were found to follow a 5- to 6-year cycle, but this pattern disappeared as incidence decreased (*7*). Annual scarlet fever notifications in Hong Kong remained low during 2001–2010 (<4 cases/100,000 population) and did not demonstrate any apparent long-term pattern. The recent increase in scarlet fever notifications might be attributable to antigenic drift, increase in virulence of GAS (*8*), or increased circulation of GAS. However, other than mandatory notification of medically attended case-patients, systematic laboratory testing of GAS isolates was not conducted in Hong Kong, and these possibilities could not be further investigated.

Notifications of scarlet fever usually peak during December–March in Hong Kong, but the outbreak in 2011 peaked in June (Figure, panel B). The rise in scarlet fever cases in Guangdong Province and Macau slightly preceded that in Hong Kong; cases in Guangdong peaked in April (Figure, panel A). Maximum cross-correlations between spline-interpolated weekly scarlet fever notifications in Guangdong and Macau and those in Hong Kong were found at 1- and 2-week lags, respectively (ρ = 0.45 and 0.58) (Technical Appendix).

In 2011, scarlet fever notification rates were elevated in all 4 regions of Hong Kong: New Territories East, New Territories West, Kowloon, and Hong Kong Island at 27.2, 21.7, 18.9, and 19.6 cases per 100,000 population, respectively. However, a distinctly higher proportion of imported cases before July 2011 (12 of 14, p value for exact binomial test = 0.01) were notified in New Territories East and New Territories West, where the main border crossings to mainland China are located. This finding suggests a link to the outbreak in Guangdong in these regions during the early phase of the local outbreak.

We estimated the instantaneous reproduction number (*R_t_*), which measures the time-dependent frequency of transmission per single primary case (Technical Appendix) (*9*). An *R_t_* consistently >1 would indicate sustained local transmission. We estimated *R*_t_ on the basis of the daily scarlet fever notification data in different periods, adjusted for imported cases. For 19 cases (1.2% of all cases), we could not determine whether infection was local or imported. We estimated *R*_t_ in 2 different ways: either by assuming that all of these cases were local or by assuming that they all were imported, to represent possible extreme values of *R*_t_. *R*_t_ fluctuated between 0.6 and 2.0 and was consistently >1 from mid-May through the end of June. *R*_t_ fell quickly to <1 beginning in early July after 2 fatal scarlet fever cases were reported on May 29 and June 21, which raised widespread concern in the community (Figure, panel C). Heightened surveillance, publicity, health education to the public (Technical Appendix) were implemented by the Centre for Health Protection in early June and could have contributed to the reduction in transmissibility. The health education measures included guidance on prevention and control measures, such as updates of antimicrobial drug resistance profile of GAS issued to all doctors and strengthening reporting of scarlet fever cases by child care centers and schools for prompt epidemiologic investigations.

In summary, we analyzed the notification data of scarlet fever and investigated spatiotemporal spreading patterns of the disease with certain time lags in Hong Kong, Macau, and Guangdong. The estimated *R*_t_ in 2011 indicated the potential for local transmission and persistence. Such a borderless spread indicates a critical need to enhance cross-border communication and timely sharing of epidemic information so that future disease control efforts can be made at multiple geographic levels.

Technical Appendix Statistical methods and discussion of impact of public notification of disease transmission, scarlet fever outbreak, Hong Kong, 2011.
