# Supplementary material for: Scarlet Fever Outbreak, Hong Kong, 2011
Source: Emerg Infect Dis. 2012 Oct;18(10):1700–2. doi: 10.3201/eid1810.120062 (PMC3471616; doi:10.3201/eid1810.120062)
Supplement: Technical Appendix — Statistical methods and discussion of impact of public notification of disease transmission, scarlet fever outbreak, Hong Kong, 2011. [file 12-0062-Techapp-s1.pdf]

Article DOI: http://dx.doi.org/10.3201/eid1810.120062

Scarlet Fever Outbreak, Hong Kong, 2011

Technical Appendix

Statistical Methods and Discussion of Impact of Public Notification on Disease Transmission

Calculation of Cross-Correlation between Notification Data

Monthly scarlet fever notifications in Guangdong and Macau were cumulated from December 2010 to December 2011 and then smoothed using cubic spline assuming time points at 15th of each month. Weekly cumulative notifications was estimated by interpolation based on the spline function, which was then differenced to obtain weekly estimated number of notifications from week starting January 16, 2011, to week starting December 19, 2011. Cross-correlations were then calculated between estimated weekly number of notifications in Guangdong and Macau versus actual weekly notifications in Hong Kong. Maximum correlation among estimated weekly Guangdong and Macau scarlet fever notifications with different lags versus notifications in Hong Kong were identified.

Estimation of the Instantaneous Reproduction Number R_t

Let j_t be the incidence of infection within Hong Kong on day *t* (i.e., the number of new local cases), and similarly, let i_t be the incidence of imported cases on day *t*. We describe the evolution of the incidence within Hong Kong over time by employing the following renewal process (*1*):

$$j_t = R_t \sum_{\tau=1}^{t} (j_{t-\tau} + i_{t-\tau}) g_\tau \qquad \text{[E1]}$$

where R_t is the instantaneous reproduction number, which is interpreted as the average number of secondary cases on day *t* produced by a single primary case, and g_τ is the probability mass function of the generation time of length τ days. The equation [E1] describes the process of secondary transmissions within Hong Kong that are caused by local and imported cases infected in the past. The right-hand side of [E1] uses the sum of local and imported cases, i.e., $(j_{t-\tau} + i_{t-\tau})$,

because their differential roles of secondary transmission are not explicitly separable. Using the cumulative distribution of a gamma-distributed generation time, G(s), with the mean 14.0 days and standard deviation 4.9 days (*2*), and truncating the distribution at s_{max}=30 days, the discrete function g_s was calculated as

$$g_s \Rightarrow \frac{G(s)-G(s-1)}{G(s_{\max})}$$

for $s > 0$.

Assuming that observation of j_t is sufficiently characterized by a Poisson distribution, the likelihood function, which is required to estimate R_t, is proportional to

$$\prod_t j_t^{Obs_t} \exp(-j_t) \qquad [E2]$$

where Obs_t is the observed number of local cases on day t. When incorporating the influence of imported cases on the transmission dynamics in equation [E1], we ignored the time elapsed from infection of imported cases to their entry into Hong Kong due to the absence of additional datasets to support more realistic assumptions. To avoid spurious estimates of R_t due to noise, we employed a step function, especially a weekly constant model, to parameterize R_t. The maximum likelihood estimates of R_t were obtained by minimizing the negative logarithm of [E2].

Supplementary Discussion

In the main text, we have shown that the instantaneous reproduction number, R_t fluctuated below and above 1. This is not surprising, because scarlet fever has been, even at low frequency, continuously seen in Hong Kong. However, when there was a surge of notifications from February to April 2011, R_t still fluctuated, indicating that the local transmission may not have been so intense. Moreover, even though the estimates of R_t were above 1 from late May to June 2011, it should also be noted that this time period was accompanied by intense media coverage and the reporting coverage of scarlet fever is likely to have been greatly improved, which could plausibly explain the observed pattern and still suggests that R_t of scarlet fever in Hong Kong is around 1. Of course, $R_t > 1$ from late May to June 2011 indicates that the population in Hong Kong has been susceptible to the epidemic, but the disease may not be so transmissible. The publicity of the second death associated with scarlet fever in late June, along

with guidance on prevention and control measures to doctors, schools and institutions concerning updates of scarlet fever activity, suggested antibiotic treatment with respect to antibiotic resistance pattern and recommended sick leave duration for children, distribution of health education materials to the public concerning basic clinical and epidemiological knowledge and personal protection against scarlet fever, in various forms such as pamphlets, posters, stickers, TV and radio announcement further elevated concern and alert in the general population, which resulted in reduced transmission efficiency as reflected by the sharp drop of R_t in early July. There are two important implications for the epidemic which is likely fueled by continued importations. First, epidemiological monitoring at multi-geographic levels and its cross-border sharing are essential, as the epidemic dynamics are largely governed by large-scale spatial interactions. Second, public health control measures may be beneficial given that R_t declined below 1 in July 2011, but radical control measure may only reduce local transmissions instantaneously, and rather, long-term epidemic trend may be more likely regulated by introductions of cases from different locations.

References

1. Nishiura H, Chowell G. The effective reproduction number as a prelude to statistical estimation of time-dependent epidemic trends. In: Mathematical and statistical estimation approaches in epidemiology. New York: Springer; 2009. p. 103–21.
2. Whitelegge A. The period of infection in scarlet fever. Lancet. 1889;133(3410):14–5. http://dx.doi.org/10.1016/S0140-6736(02)04951-6
